# A Novel Non-contact Heart Rate Monitor Using Impulse-Radio Ultra-Wideband (IR-UWB) Radar Technology

**DOI:** 10.1038/s41598-018-31411-8

**Published:** 2018-08-29

**Authors:** Yonggu Lee, Jun-Young Park, Yeon-Woo Choi, Hyun-Kyung Park, Seok-Hyun Cho, Sung Ho Cho, Young-Hyo Lim

**Affiliations:** 1 Division of Cardiology, Department of Internal medicine, College of Medicine, Hanyang University, Seoul, Republic of Korea; 2 Department of Electronics and Computer Engineering, College of Engineering, Hanyang University, Seoul, Republic of Korea; 3 Department of Pediatrics, College of Medicine, Hanyang University, Seoul, Republic of Korea; 4 Department of Otorhinolaryngology, College of Medicine, Hanyang University, Seoul, Republic of Korea

**Keywords:** Cardiac device therapy, Physical examination, Biomedical engineering

## Abstract

We discovered that impulse-radio ultra-wideband (IR-UWB) radar could recognize cardiac motions in a non-contact fashion. Therefore, we measured the heart rate (HR) and rhythms using an IR-UWB radar sensor and evaluated the validity and reliability of the measurements in comparison to electrocardiography. The heart beats were measured in 6 healthy volunteers (18 samples) with normal sinus rhythm (NSR) and 16 patients (36 samples) with atrial fibrillation (AF) using both an IR-UWB radar sensor and electrocardiography simultaneously. The participants hold their breath for 20 seconds during the data acquisition. In subjects with NSR, there was excellent agreement of HR (intraclass correlation coefficient [ICC] 0.856), average R-R interval (ICC 0.997) and individual R-R intervals between the two methods (ICC 0.803). In subjects with AF, HR (ICC 0.871) and average R-R interval (ICC 0.925) from the radar sensor also agreed well with those from electrocardiography, though there was a small disagreement in the individual R-R intervals between the two methods (ICC 0.697). The rhythms computed by the signal-processing algorithm showed good agreement between the two methods (Cohen’s Kappa 0.922). The IR-UWB radar sensor is precise and accurate for assessing HR and rhythms in a non-contact fashion.

## Introduction

Recently, the usefulness of continuous vital sign monitoring technologies has been recognized for various medical purposes, including intensive care for critically ill patients and protection of the elderly and infants from sudden cardiac death at home or in health institutions^1–4^. All continuous heart beat monitoring devices currently available in the medical field, including conventional electrocardiography (ECG), pulse oximetry and innovative wearable devices, require physical contact between the skin and electrodes, infrared sensors or pressure sensors. Physical contact in the use of those devices not only limits the mobility of patients but may also cause local problems on the skin and the spread of contagious infections between patients^4–6^. In places such as intensive care units and low-birth-weight newborn care units, a simple skin defect initiated when electrodes detach from an unconscious patient may serve as a route of entry for serious infections. Continuous heart beat monitoring for a long duration also require continuous replacements of expendables. It would be valuable if heart monitoring could be achieved accurately without physical contact. Impulse-radio ultra-wideband (IR-UWB) radar may deliver on all counts.

Through emitting a band of radio waves in the electromagnetic spectrum and receiving the reflected waves from an object, the radar system detects movements of objects and keeps track of the moving objects with high precision. Ultra-wideband (UWB) systems employ radio waves occupying a frequency band of >500 MHz, or 25% of the fractional bandwidth. Because of the wide bandwidth, UWB systems could have many advantages, including high resolution, multipath resistance, good penetration power and simple hardware structure^7^. Since 2007, when UWB standardization was established by the standardization group IEEE 802.15.4a^8^, much research has been conducted on the standardized protocol. The IR-UWB radar is a system that uses UWB radar as a form of impulse waves. Because it uses impulse waves of very short durations, it can deliver a high resolution that enables the measurement of the motions of human bodies. This has been studied widely in the field of security, disaster prevention and crowdedness measurement^9,10^. Moreover, because of its good penetrating power and high resolution, IR-UWB radar is speculated to be able to detect the motions of internal organs such as the heart outside of the body from a distance.

In this study, we investigated whether an IR-UWB radar sensor could recognize cardiac motion and whether we could monitor the heart rate using IR-UWB radar accurately compared to ECG in subjects with normal sinus rhythm (NSR) and in those with atrial fibrillation (AF).

## Subjects and Methods

### Subjects

To validate the accuracy and reliability of IR-UWB radar measurements for the heart in both regular and irregular heart rhythms, we conducted this study with 6 healthy participants with NSR and 16 patients with AF. All the subjects voluntarily participated in this study. Demographic and anthropometric information was collected from all participants before radar measurements. All participants underwent ECG to determine the baseline rhythm before entering the study. The healthy volunteer was defined as a participant with NSR who had no known cardiovascular diseases and the patient with AF was defined as a participant with AF who had previously been documented with either persistent or permanent AF and receiving medical therapy for rate control. Written informed consent was obtained from all participants before they entered the study. The Institutional Review Board of Hanyang University Hospital reviewed and approved the study protocol and monitored the study processes. All methods were performed in accordance with relevant guidelines and regulations (IRB No. 2017-05-004-001).

### Data acquisition from IR-UWB radar

**Figure 1:**
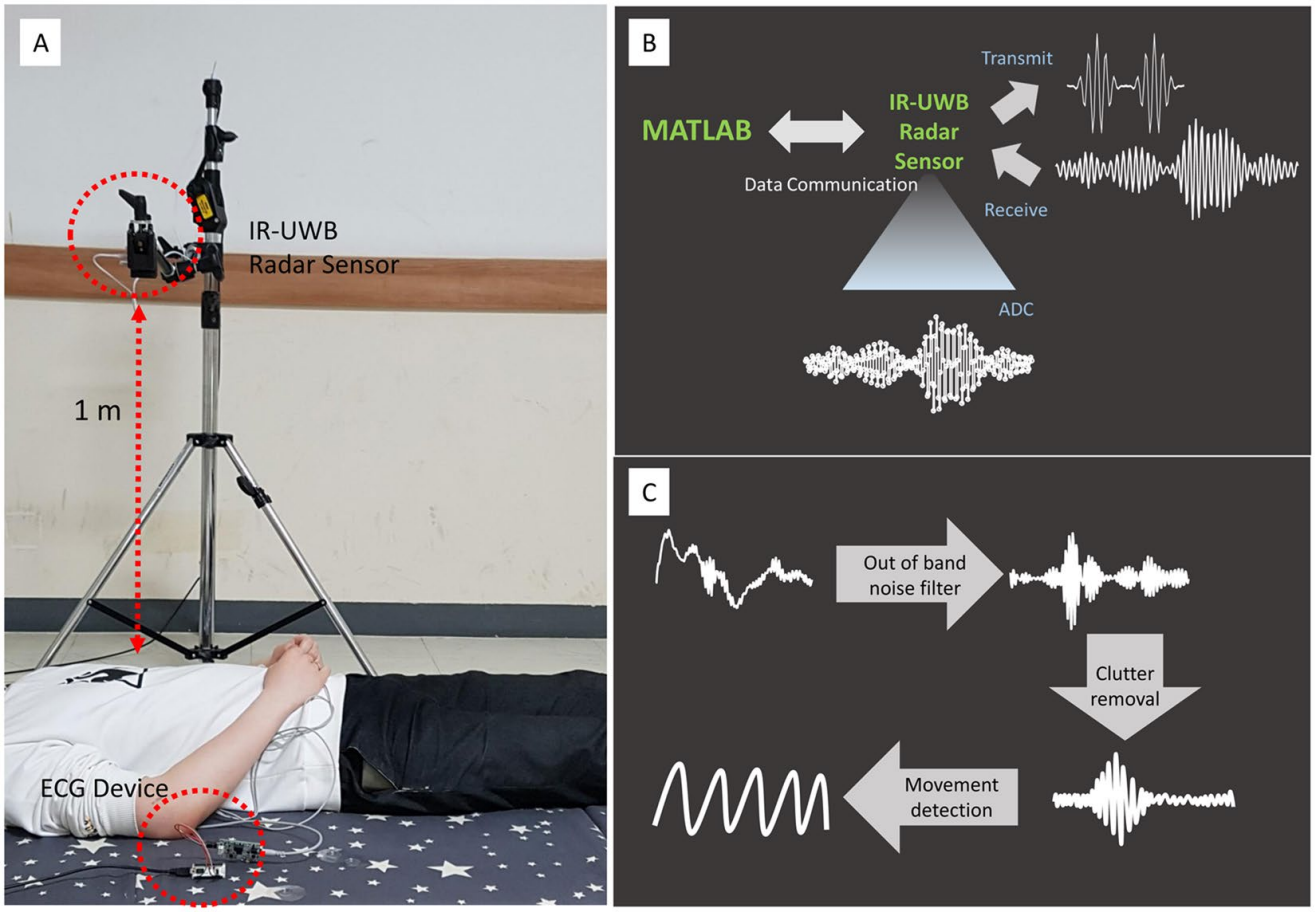
Acquisition and processing of the IR-UWB radar signals from the heart. (**A**) The experiment was conducted with participants whose clothes were on after 5–10 minutes’ rest. To measure the heart signals only, the participants were told to hold their breath for 20 seconds. During the breath-holding, the IR-UWB radar and ECG were measured simultaneously. (**B**) The IR-UWB radar system transmitted radiofrequency pulse waves and received signals reflected from an object. Analogue signals received from the object were digitalized by an ADC chip embedded in the radar device. The digital data obtained from the radar were sent to MATLAB for further processing. (**C**) The raw signals passed through a bandpass filter to cancel noise, then were processed to separate clutter signals from target signals. By tracing the target signals during breath-holding, the heart signals were obtained.

Participants stayed at rest for 5 minutes until they reached a comfortable condition before the data acquisition using the IR-UWB radar. When a comfortable condition was not achieved after 5 minutes, participants took an additional 5-minute rest before the measurement. The data were acquired in the supine position with clothes on. Heart beats were simultaneously measured from the IR-UWB radar and ECG so that the data recorded from the two devices were synchronized. Participants were told to hold their breath for 15–20 seconds during the measurements, to minimize the noises from the chest wall during respiration. Because it took a few seconds for the radar device to acquire clear data without respiratory noise after the participant started holding their breath, the data acquired for the last 10 seconds were used in the analysis. The heart beats were measured 3 times per participant. In some patients with AF who had difficulty holding their breath, the data acquisition was conducted only 1–2 times. The detailed settings for the radar measurement are depicted in Fig. 1A. A commercially available IR-UWB radar device, X4M06 (Novelda, Oslo, Norway) was used to send and collect the radar signals to and from the heart. The radar device has been certified by both Korea Communications Commission and Federal Communications Commission. (The certification can be found in the following website: https://www.xethru.com/community/resources/categories/datasheets.5/). The radiation power of the radar device was 68.85 μW, and in the central frequency of 8.7 GHz, the penetration depths where the power decreases to 1/*e* of the original power was estimated to 1.6 mm for skin and 1.4 mm for muscles. The detailed processes of the data acquisition are described in Fig. 1B. For acquiring, processing and storing the data from ECG and the radar, MATLAB (MathWorks, New York, MA, USA) was used.

### ECG measurement

A single bipolar lead ECG was measured simultaneously with the radar using an ECG device, PSL-iECG2 (PhyioLab, Pusan, Republic of Korea). The electrodes for the ECG device were placed on both wrists and the left elbow joint, and the data from the limb lead II were obtained. The data from ECG were synchronized and stored with the radar data using MATLAB.

### Radar data processing

IR-UWB radar devices transmit signals and receive them when they are reflected by obstacles. In a radar system, the received analog signals are sampled and quantized by an analog-to-digital converter (ADC) to become digital data. The digital data were transferred to MATLAB for further data processing. Because the received signals contained various noises outside the frequency range, we canceled the noises using the bandpass filter. These raw radar signals contained various unwanted background noises within the radar bandwidth, called clutter signals, along with the target signals for heart movements (Fig. 1C). The target signals were obtained by subtracting the clutter signals from the raw data by the following algorithm using a moving-average method.

$${B}_{k}=(1-\alpha ){B}_{k-1}+\alpha {x}_{k}$$

$${S}_{k}={x}_{k}-{B}_{k}$$

where *B*_*k*_ is the k^th^ clutter signal, *x*_*k*_ is the k^th^ received signal, *S*_*k*_ is the k^th^ target signal, and *α* is the moving-average weight.

Stationary clutter signals should be identical when a moving average is applied, whereas moving target signals should vary with time. By subtracting the stationary clutter signals from the raw data, target signals could be obtained. Because the only movement that could be detected from a participant was the movement of the heart, when the participant held their breath, we obtained a waveform representing heart beats through the processes described above. The peak points of the waveforms were automatically recognized using our own algorithm, and the intervals between the neighboring peak points were converted to R-R intervals.

### Arrhythmia detection

To recognize AF automatically, R-R interval variations were analyzed using 3 criteria:

1. Normalized maximum difference
  {Max(RR)-Min(RR)}/ Mean(RR)> 0.2
2. Coefficient of variance of RR interval^11^
  SD(RR)/Mean(RR) >0.1
3. Normalized absolute deviation^12^

Mean[{RR-Mean(RR)}/Mean(RR)] >0.05

Where Max is the maximum value, Min is the minimal value, Mean is the mean value, SD is the standard deviation and RR is the R-R interval. The cut-off values for the criteria were set to recognize all measurements of NSR in ECG. AF was recognized when all 3 criteria were satisfied.

As an alternative to the R-R interval variation analysis, the frequency domains of the radar signals were analyzed using the method of short-time Fourier transform, which transforms the changes in frequency into changes in time. Because R-R intervals in patients with AF vary over time, AF could be recognized using this transformation. The frequencies obtained 20 times per second were averaged in a 3-second window, and the signal intensities of the frequencies were color-coded and plotted against time for 7 seconds in a 2-dimensional spectrograph of the power spectral density. The variation in the frequency of the peak signal intensity was calculated at every measurement, and the maximum frequency variation was used to determine the presence of AF.

### Statistical analysis

Continuous data, including age, height, weight, heart rate and R-R intervals, are presented as the mean ± SD. The agreements of HR, average R-R interval and individual R-R intervals between ECG and radar were evaluated using the intraclass correlation coefficient R (ICCR) and Bland-Altman plots with 2.5% and 97.5% limit of agreements. The agreement in arrhythmia recognition between ECG and radar was assessed using Cohen’s kappa test. In AF recognition using the R-R interval variation analysis, the diagnostic performance of the radar was evaluated against the rhythms determined by ECG. In the frequency domain analysis, the cut-off point for the maximum frequency variation of the peak signal intensity was decided at the maximum Youden’s J index in a receiver operating characteristic curve analysis. Using the cut-off point, the presence of AF was automatically determined. All statistical analyses were performed using the statistical software R version 3.4.0 and its packages ICC, MethComp, descr and psych. All *p*-values < 0.05 were considered significant.

## Results

**Figure 2:**
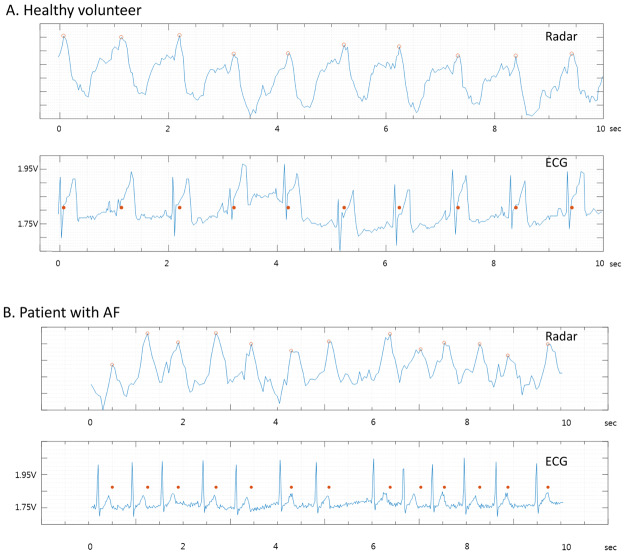
Examples of the heart signal waveforms obtained from the IR-UWB radar. The agreements between the radar and ECG were grossly excellent in both the healthy volunteers with NSR (**A**) and the patients with AF (**B**). The red dots indicate the same time points on both the radar waveform and ECG.

Baseline characteristics of participants are summarized in Table 1. The mean heart rate of the healthy volunteers and patients with AF were (70.3 ± 6.1 beats/min) and (76.7 ± 13.6 beats/min), respectively. Eighteen samples were obtained from 6 healthy volunteers (3 samples for each), whereas 36 samples were obtained from 16 patients with AF (2.25 samples for each). The waveforms obtained from IR-UWB radar and ECG in healthy volunteers and patients with AF are shown in Figs 2A and 2B.

**Table 1:** Baseline characteristics of the participants.

	Healthy volunteers	Patients with AF
	N = 6	N = 16
Age (year)	27.5 ± 2.8	70.8 ± 9.2
Sex (male)	6 (100%)	13 (81%)
Height (cm)	177.3 ± 3.3	165.6 ± 8.5
Weight (kg)	69.1 ± 10.5	67.4 ± 11.7
Body mass index (kg/m^2^)	21.9 ± 2.5	24.5 ± 3.4
Systolic blood pressure (mmHg)	—	126.1 ± 10.3
Diastolic blood pressure (mmHg)	—	75.2 ± 8.6
Heart rate (beats/minute)	70.3 ± 6.1	76.7 ± 13.6
Underlying diseases		
Hypertension	—	14 (88%)
Diabetes	—	7 (44%)
Coronary artery diseases	—	4 (25%)
Congestive heart failure	—	5 (31%)
Cerebrovascular disease	—	5 (31%)
Chronic kidney disease	—	2 (13%)

Data are presented as the mean ± SD or N (%).

**Figure 3:**
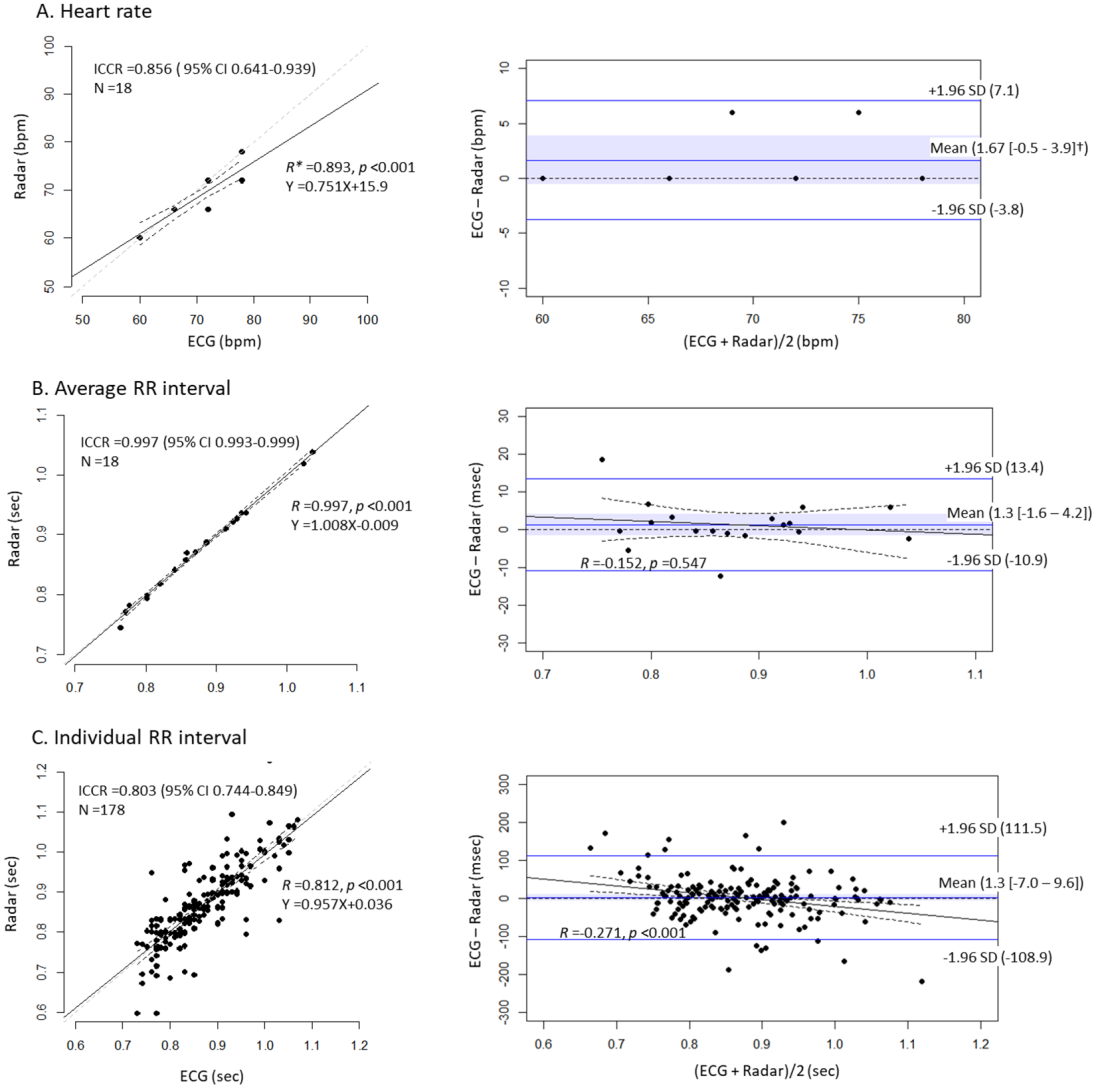
Comparison of heart rate and RR intervals between ECG and radar in the healthy volunteers with NSR Heart rate (**A**), average RR interval (**B**) and individual RR intervals (**C**) measured with IR-UWB radar highly agreed with those measured with ECG. The mean biases were not significant in any of the 3 parameters. There was a proportional difference in the individual RR intervals between ECG and radar. *All *Rs* were derived from Pearson’s correlation tests. ^†^The blue ribbons and the numbers inside the brackets indicate 95% confidence intervals of the mean bias between ECG and radar measurements. ECG, electrocardiography; ICCR, intraclass correlation coefficient R; SD, standard deviation.

In the healthy volunteers with normal sinus rhythm, we found that HR, average R-R interval and individual R-R interval measured in the radar showed high agreements with those measured in ECG. The average R-R interval showed the highest agreement level, while the individual R-R interval showed lowest agreement level between the 2 measurements. The 95% confidence intervals (CIs) of the mean biases included the lines of equality in all 3 parameters, indicating no significant biases between the ECG and radar parameters. However, the absolute values of the limits of agreements were all >100 milliseconds, and there was a negative proportional difference between ECG and radar in the measurements of the individual R-R interval (Fig. 3).

**Figure 4:**
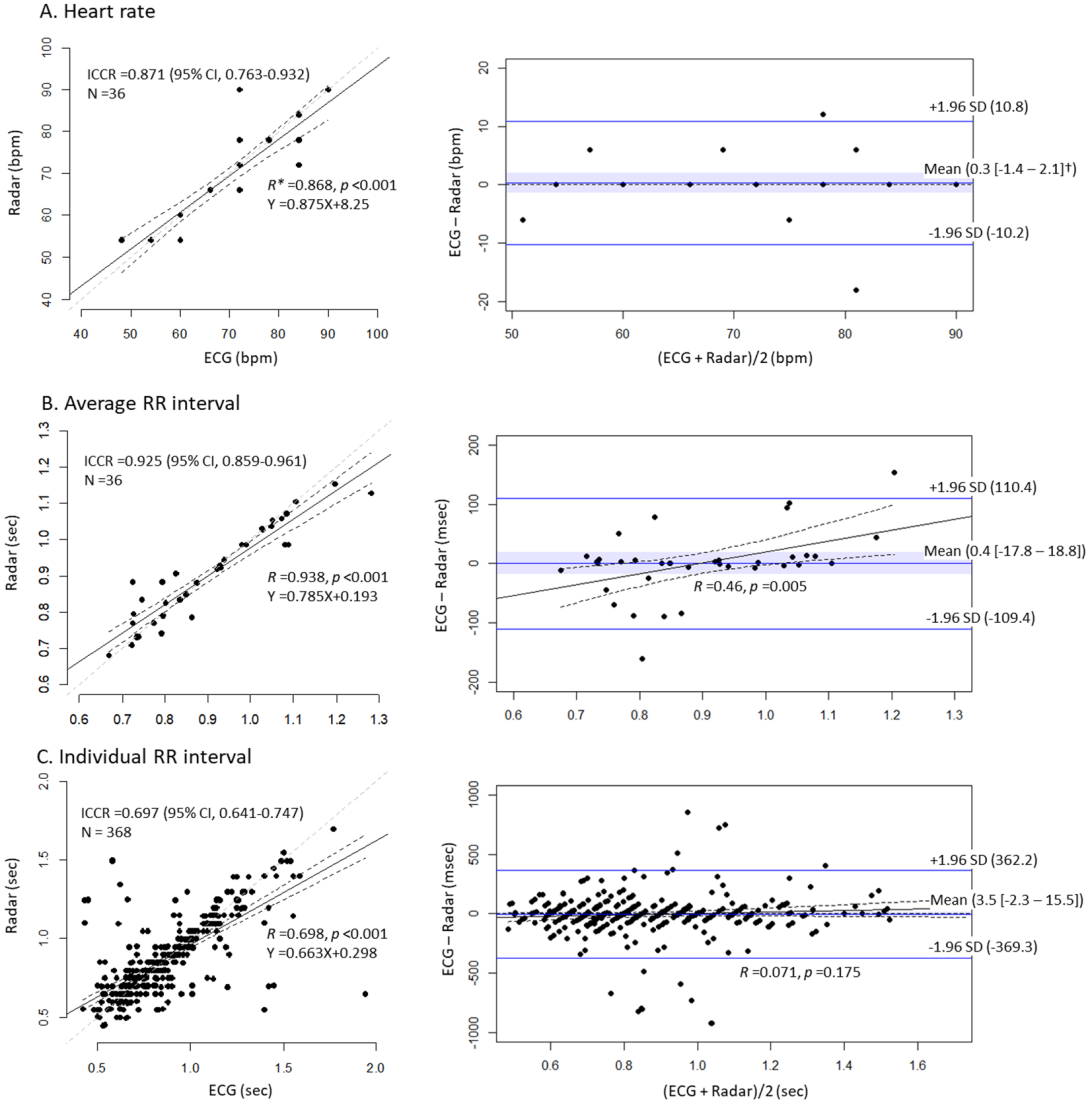
Comparison of heart rate and RR intervals between ECG and the radar in patients with AF. Heart rate (**A**) and average RR interval (**B**) showed high agreement, while individual RR intervals (**C**) showed moderate agreement. The mean biases were not significant in any of the 3 parameters. There was a proportional difference in the average RR interval between ECG and radar. *All *Rs* were derived from Pearson’s correlation tests. ^†^The blue ribbons and the numbers inside the brackets indicate 95% confidence intervals of the mean bias between ECG and radar measurements. ECG, electrocardiography; ICCR, intraclass correlation coefficient R; SD, standard deviation.

In the patients with AF or other diseases, HR and average R-R interval measured in the radar also highly agreed with those measured in ECG, and the same was observed in healthy volunteers, whereas individual R-R interval showed a slightly lower agreement level. The average R-R interval showed the highest agreement level, while the individual R-R interval showed the lowest, and the 95% CIs of the mean biases included the lines of equality in all 3 parameters in both groups of patients. However, the absolute values of the limit of agreements were >350 milliseconds in the measurements of the individual R-R intervals. There was a positive proportional difference between ECG and radar measurements of the average R-R interval (Fig. 4).

**Figure 5:**
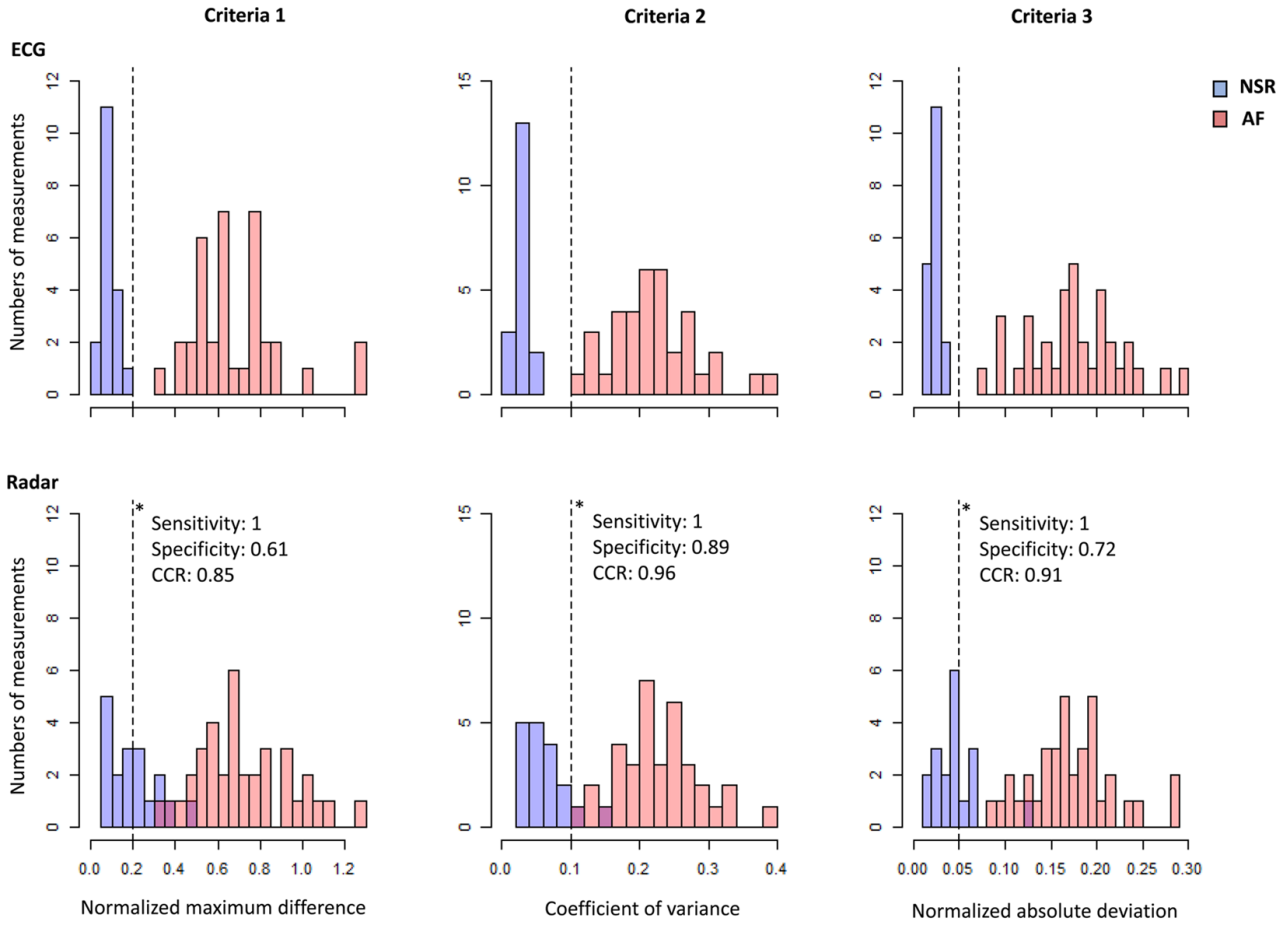
Distribution of the 3 parameters for RR interval differences in the healthy volunteers and patients with AF. Criteria 1: {Max(RR)-Min(RR)}/Mean(RR) >0.2. Criteria 2: SD(RR)/Mean(RR) >0.1. Criteria 3: Mean[{RR-Mean(RR)}/Mean(RR)] >0.05. The cut-off value of each criterion was set to include AF maximally and to exclude all sinus rhythms in ECG measurements. AF was recognized automatically when all 3 criteria were satisfied. The same criteria were applied to the radar measurements to automatically detect AF using radar. *Diagnostic performance at each cut-off value.

Using the 3 criteria of AF, we automatically determined the presence or absence of AF in healthy volunteers and patients with AF. In the ECG measurements, all 3 criteria completely differentiated sinus rhythm from AF, whereas 8 (44%), 2 (11%) and 5 (28%) samples of sinus rhythm did not satisfy the criteria in the radar measurements (Fig. 5). When a combination of the 3 criteria was used, ECG differentiated sinus rhythm from AF correctly in all samples, whereas radar correctly classified 52 of 54 samples and misclassified 2 samples of sinus rhythm into AF (correct classification rate, 96.2%). The Cohen’s kappa of 0.91 indicated a high agreement level between the classifications using ECG and radar. The sensitivity and specificity of AF diagnosis using radar were 100% and 89%, respectively (Table 2).

**Table 2:** Recognition of AF using IR-UWB Radar.

		ECG		
		AF (N = 36)	NSR (N = 18)	Total
Radar (R-R interval analysis)	AF	36	2	38
*K* = 0.91 (0.80–1.00)*	NSR	0	16	16
		Sensitivity = 1.00	Specificity = 0.89	CCR = 0.958
Radar (spectrography)	AF	36	3	39
*K* = 0.87 (0.73–1.00)*	NSR	0	15	15
		Sensitivity = 1.00	Specificity = 0.83	CCR = 0.944

*Cohen’s kappa (95% confidence interval).

AF, atrial fibrillation; NSR, normal sinus rhythm; ECG, electrocardiography; CCR, correct classification rate.

**Figure 6:**
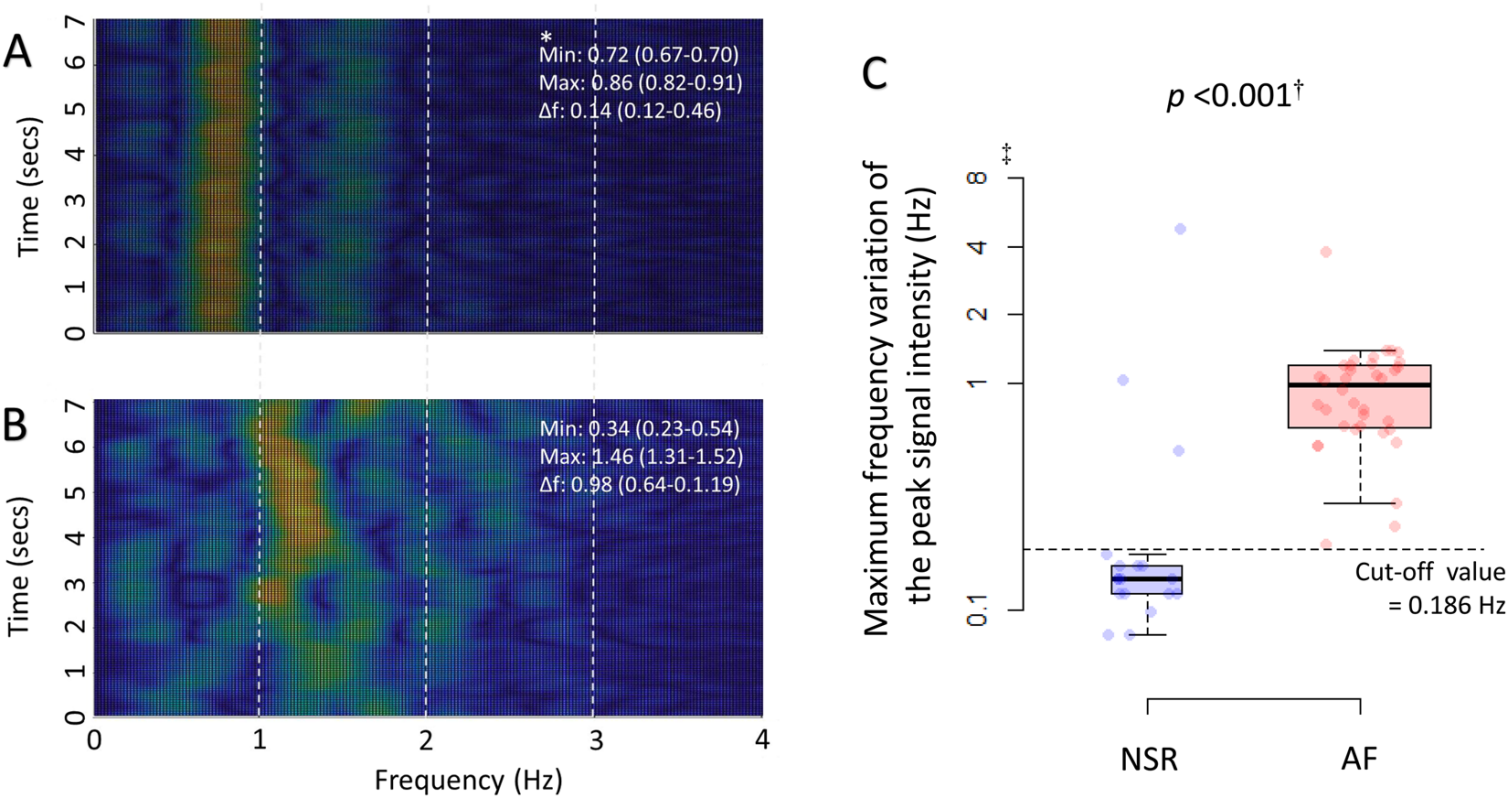
Detection of AF using the frequency domain of the radar signals. The signal intensities of the frequencies in the radar raw data were color-coded and plotted on spectrograms of power spectral density in all participants using the method of short-time Fourier transform. There were distinctive patterns in the spectrograms obtained from the healthy volunteers with NSR (**A**) vs. those obtained from the patients with AF (**B**). The frequency variation of the peak signal intensity was wider in the patients with AF than those with NSR. The maximum variation in the frequency of the peak signal intensity was significantly higher in the patients with AF than in the healthy volunteers with NSR (**C**). *The data are median (interquartile range). ^†^Mann-Whitney U test. ^‡^The Y-axis is presented on a log scale due to the skewedness of the frequency variation. NSR, normal sinus rhythm; AF, atrial fibrillation; Min, minimal frequency of the peak signal intensity; Max, maximal frequency of the peak signal intensity; Δf: maximum frequency variation of the peak signal intensity.

The spectrograms in patients with AF showed a distinctive pattern compared with those in healthy volunteers with NSR (Fig. 6). The frequency of the peak signal intensity did not vary over time in healthy volunteers with NSR, whereas the frequency continuously shifted over time in patients with AF. When the cut-off point for the maximum differences in the frequency was determined to 0.5 Hz by the receiver operating characteristic curve analysis (C-statistic: 0.894 [95% confidence interval 0.772–1.000]; Supplementary Fig. S1), the radar showed a slightly lower yet good agreement level with ECG and diagnostic performances for AF, compared with the R-R interval analysis (Table 2).

## Discussion

In this study, we found that IR-UWB radar can recognize heart beats with high precision without physical contact. Heart rate and R-R interval measured using the IR-UWB radar agreed well with those measured using ECG in both NSR and AF patients. Our results also show that IR-UWB radar signals from the heart can accurately distinguish atrial fibrillation from NSR.

Non-invasive heart monitoring modalities, including ECG and pulse oximetry, are widely used in daily clinical practice. However, to date, there is no established modality to monitor heart beats without physical contact. Non-contact heart monitors would have many advantages over conventional heart monitors, which limit the patient’s mobility, cause local problems associated with electrode attachment such as skin eruptions, abrasion and bruise, transmit contagious diseases and require occasional replacement if the device will be in prolonged uses^5,13^. A non-contact heart monitor using IR-UWB radar could be a valuable tool in various clinical fields if its accuracy and reliability are sufficiently high. The IR-UWB radar employs pulses of electromagnetic waves which have never been exploited for a test modality in clinical fields. Because the level of energy that the radar devices deliver to tissues is less than 0.01% of that emitted by an ordinary 5 GHz WIFI device, which is typically 1 W, the radar measurement would be safe even for patients undergoing prolonged exposure. For the first time, we found that the IR-UWB radar could measure the fine movements of the heart, and we validated the accuracy and reliability of heart rate and R-R interval measured by the radar. Recently, wearable heart rate monitoring devices have been developed and applied in both clinical and non-clinical fields^14–18^. The ICCRs between heart rates computed by currently available wearable devices and those from ECG ranged between 0.80 and 0.95^18^, which are comparable to our results. Given that the radar sensor measured heart rates in a non-contact fashion and we included patients with AF whose R-R intervals are irregular, the accuracy level of the radar measurements in our results is quite notable.

In our results, the percentage mean error (2.3% vs. 0.2%) and ICCR (0.856 vs. 0.997) of heart rate were much larger than those of average R-R interval in healthy volunteers with NSR, although a heart rate is only proportional to the reciprocal of the average R-R interval. This is because we used heart rates measured for only 10 seconds in the analysis. The measurement errors in the heart rates for 1 minute could be multiplied by a factor of 6. In contrast, differences between percentage mean error (0.5% vs. 0.3%) and ICCR (0.871 vs. 0.925) of heart rate and those of average R-R interval decreased in patients with AF, which may be related to the increased number of samples and decreased accuracy of R-R interval measured using the radar in AF. Radar tended to measure average R-R intervals as longer in patients with AF, when heart rates were higher. Because the raw radar data contained various noises that were difficult to eliminate completely, even with breath-holding, filtering and sophisticated algorithmic canceling, small peaks and troughs on the radar waveform remained. R-R intervals were recognized between the neighboring peaks on the waveform, which occasionally became indistinguishable in rapid heart rates. In contrast, a prominent noise peak could be more easily recognized as a heart beat in slow heart rates. These considerations may explain the proportional bias of the average R-R interval as well as the relatively low agreement level of individual R-R interval in patients with AF, whose R-R intervals fluctuate.

There were intervals between R waves in ECG and the peaks in the radar waveform (Fig. 2). Because the peak point in a cycle of the radar waveform is when the cardiac muscle is closest to the radar sensor orthogonal to the anterior chest wall in a supine position, we think the rapid descents after the peaks in the radar waveform correspond to the beginning of left ventricular ejection and the intervals between the R wave and the peaks correspond to the isovolemic contraction time. Since the intervals between neighboring peaks in the radar waveform were recognized as R-R interval, it is important to pinpoint the peak in a single cycle of the waveform. However, in the patients with AF, a cycle of the radar waveform often contained multiple indistinguishable peaks with similar heights which may be associated with remaining noises, atrial movements or the insufficient radar sampling rate. These multiple peaks in a cycle may have also caused the lower agreement level between the radar measurements and ECG measurements in the patients with AF.

To date, several non-contact heart-monitoring technologies have been introduced. Huang *et al*. have reported that heart beat monitoring was possible without direct contact on the skin using capacitive-coupled devices integrated in the home sofa^19^. However, unlike radar, which can measures heart rate at a 1-m distance, this method requires a very close proximity between device and skin. In fact, the subjects still had to contact the sofa. The report also did not contain any data related to the accuracy or reliability. Jeger-Madiot *et al*. have reported vibrocardiography using an airborne pulse-Doppler ultrasound system that emits ultrasound pulses on the chest and recognizes the reflected waves from the heart through a microphone with clothes on^20^. Their method did not require direct contact with the device itself. However, the distance between the device and the clothes was 8 cm, which may still be considered close proximity. They also did not provide any validation data for their method. Several photoplethysmography methods have been introduced for heart rate measurement at a distance^21–24^. PhysioCam^24,25^ and Microsoft Kinect^22^ have demonstrated good accuracies in heart rate measurements using photoplethysmography methods. Unlike radar, photoplethysmography recognizes blood volume changes in the microcirculation using visible light on the exposed skin surface. In patients with rapid or irregular heartbeats, such as in those with AF, the pulsating signal intensity may not easily be distinguished by photoplethysmography because there would be little blood pressure difference to distinguish between 2 very closely neighboring heart beats^21^. Because IR-UWB radar directly measures movements of the heart, it may be more effective under those conditions than photoplethysmography.

Apparently, our radar technology in the current form could not be utilized as a continuous heart rate monitor until breath-holding during radar measurement is not necessary. However, because our radar provide not only heart rates but also signal waveforms that allow us to recognize individual heart beat durations, the current technology alone may have utilities in daily clinical practices. Through a short breath-holding, physicians could recognized heart rates and rhythm without a physical contact to the patients whose clothes are on, which would be particularly useful when physical contacts with patients should be avoided. Although we did not provide any specific data in this study, we think that the IR-UWB radar waveform also contains the information about global myocardial motions. In future, with proper validation and calibration, physicians may also be able to assess the global cardiac function of patients through a short breath-holding without contact.

### Limitation

This heart-monitoring technology using IR-UWB radar has aspects to be improved. First, the radar measured heart beats while participants were holding their breath. To use the IR-UWB radar as a non-contact heart monitoring method in clinical practice, it is necessary for the radar technology to achieve sufficient accuracy and reliability in measurement settings in which gentle motions are permitted and breath-holding is not necessary. Currently, we are conducting experiments to improve our radar signal recognition algorithms and techniques, to eventually achieve the level of sophistication in which heart rate variability can be monitored in daily life using the IR-UWB radar. Second, all the measurements were obtained in the supine position with a fixed 90-degree tangential angle between the radar device and the body. The measurement performance may change when the posture of the subject or the relative position of the radar to the body changes.

## Conclusion

Non-contact heart monitoring using the IR-UWB radar could accurately and reliably measure heart rate and R-R interval in both NSR and AF and could distinguish AF from NSR with high precision, when the respiratory movements on the chest were excluded in a resting state. Although the technology is still in its infancy and many improvements should be achieved in terms of the limited measurement conditions before it could be used clinically, it will be a useful heart-monitoring method that can overcome the difficulties of conventional heart monitors requiring physical contact with the patient.

### Electronic supplementary material

Supplementary Fig. S1
