# Supplementary material for: A Novel Non-contact Heart Rate Monitor Using Impulse-Radio Ultra-Wideband (IR-UWB) Radar Technology
Source: Sci Rep. 2018 Aug 29;8:13053. doi: 10.1038/s41598-018-31411-8 (PMC6115337; doi:10.1038/s41598-018-31411-8)
Supplement: Supplementary file 1 — Supplementary Fig. S1 [file 41598_2018_31411_MOESM1_ESM.docx]

**A Novel Non-contact Heart Rate Monitor Using Impulse-Radio Ultra-Wideband (IR-UWB) Radar Technology**

Yonggu Lee^1,†^, Jun-Young Park^2,†^, Yeon-Woo Choi^1†^, Hyun-Kyung Park^3^, Seok-Hyun Cho^4^, Sung Ho Cho^2,^*, Young-Hyo Lim^1,^*

^1^Division of Cardiology, Department of Internal medicine, College of Medicine, Hanyang University, Seoul, Republic of Korea

^2^Department of Electronics and Computer Engineering, College of Engineering, Hanyang University, Seoul, Republic of Korea

^3^Department of Pediatrics, College of Medicine, Hanyang University, Seoul, Republic of Korea

^4^Department of Otorhinolaryngology, College of Medicine, Hanyang University, Seoul, Republic of Korea

**Supplementary Data**

Supplementary Figure 1. The diagnostic performances of the maximum frequency variation of the peak radar signal intensity for the AF.


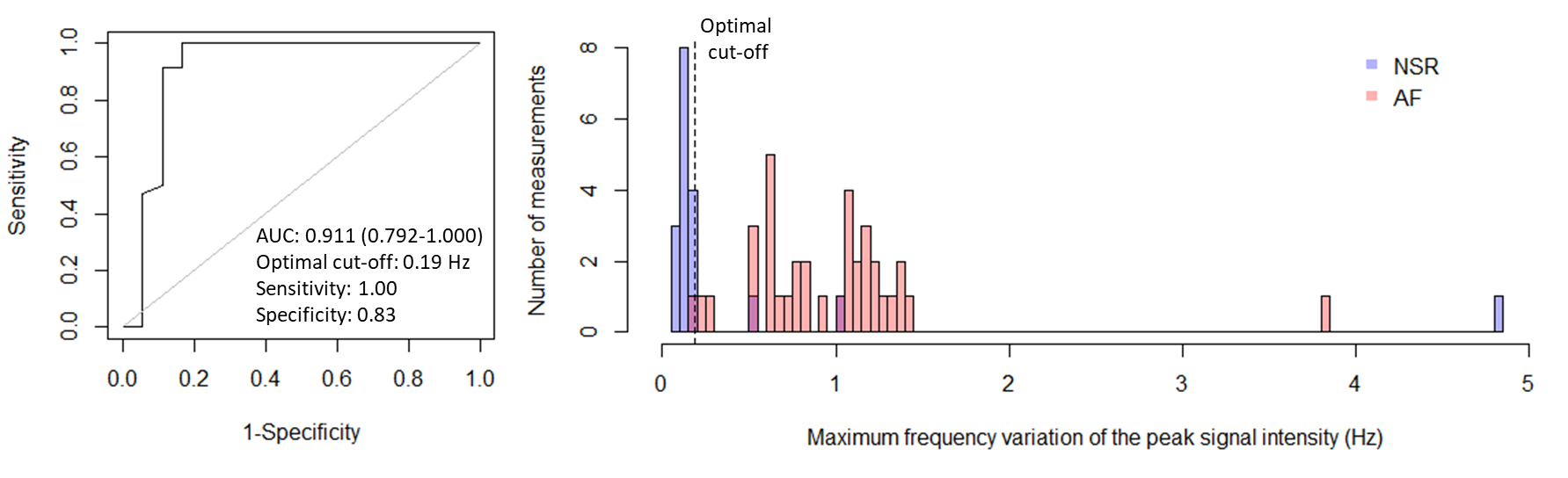


The receiver operating characteristic curve analysis showed that the maximum frequency variation of the peak radar signal intensity had good diagnostic performances with high sensitivity and specificity at the optimal cut-off value of 0.186 Hz. The histogram showed that no measurements of AF were incorrectly classified with normal sinus rhythm by the cut-off value.
